# Supplementary material for: Convergent latitudinal erosion of circadian systems in a rapidly diversifying order of fishes
Source: bioRxiv. 2025 May 31:2025.05.28.656707. Preprint. [Version 1] doi: 10.1101/2025.05.28.656707 (PMC12154703; doi:10.1101/2025.05.28.656707)
Supplement: 1 [file NIHPP2025.05.28.656707V1-supplement-1.pdf]

bioRxiv preprint doi: https://doi.org/10.1101/2025.05.28.656707; this version posted May 31, 2025. The copyright holder for this preprint (which was not certified by peer review) is the author/funder, who has granted bioRxiv a license to display the preprint in perpetuity. It is made available under aCC-BY 4.0 International license.

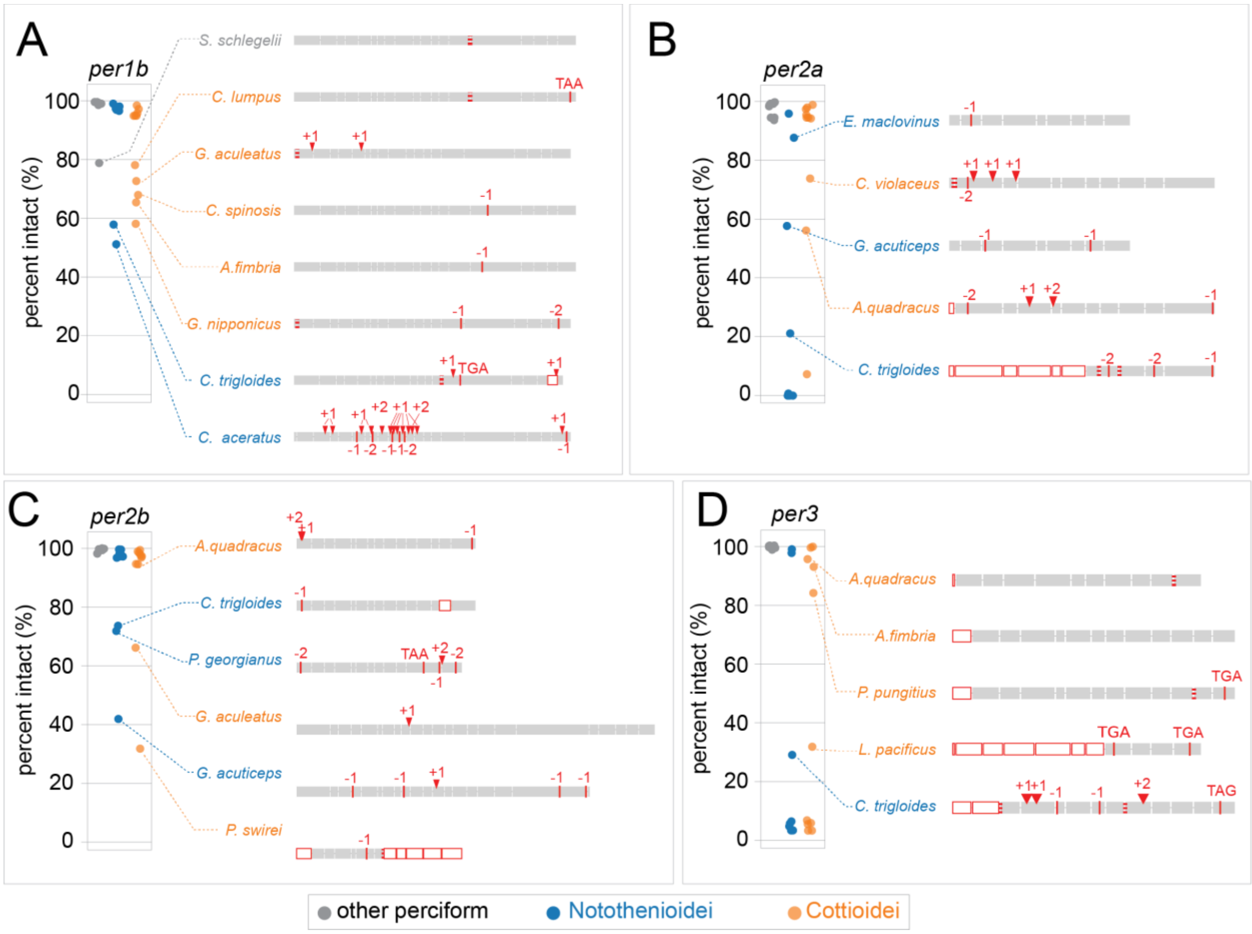


**Fig. S1 - Example mutational variants across *period* genes**. A) *per1b*, B) *per2a*, C) *per2b*, D) *per3*. Plots show the maximum percentage of intact coding sequence across all transcript isoforms annotated via pairwise genome alignments to *S. lucioperca* and *S. aurata* reference genomes. Species are grouped as Notothenioidei (blue), Cottioidei (orange), or other Perciformes (gray). Representative mutations across each exon for select species are shown to the right. Exons are shaded gray; deleted or missing exons are white with a red outline. Truncating variants are labeled by their type (e.g., frameshifts as +1 or –1), and splice site mutations are marked with red dashed lines at exon boundaries.

bioRxiv preprint doi: https://doi.org/10.1101/2025.05.28.656707; this version posted May 31, 2025. The copyright holder for this preprint (which was not certified by peer review) is the author/funder, who has granted bioRxiv a license to display the preprint in perpetuity. It is made available under aCC-BY 4.0 International license.

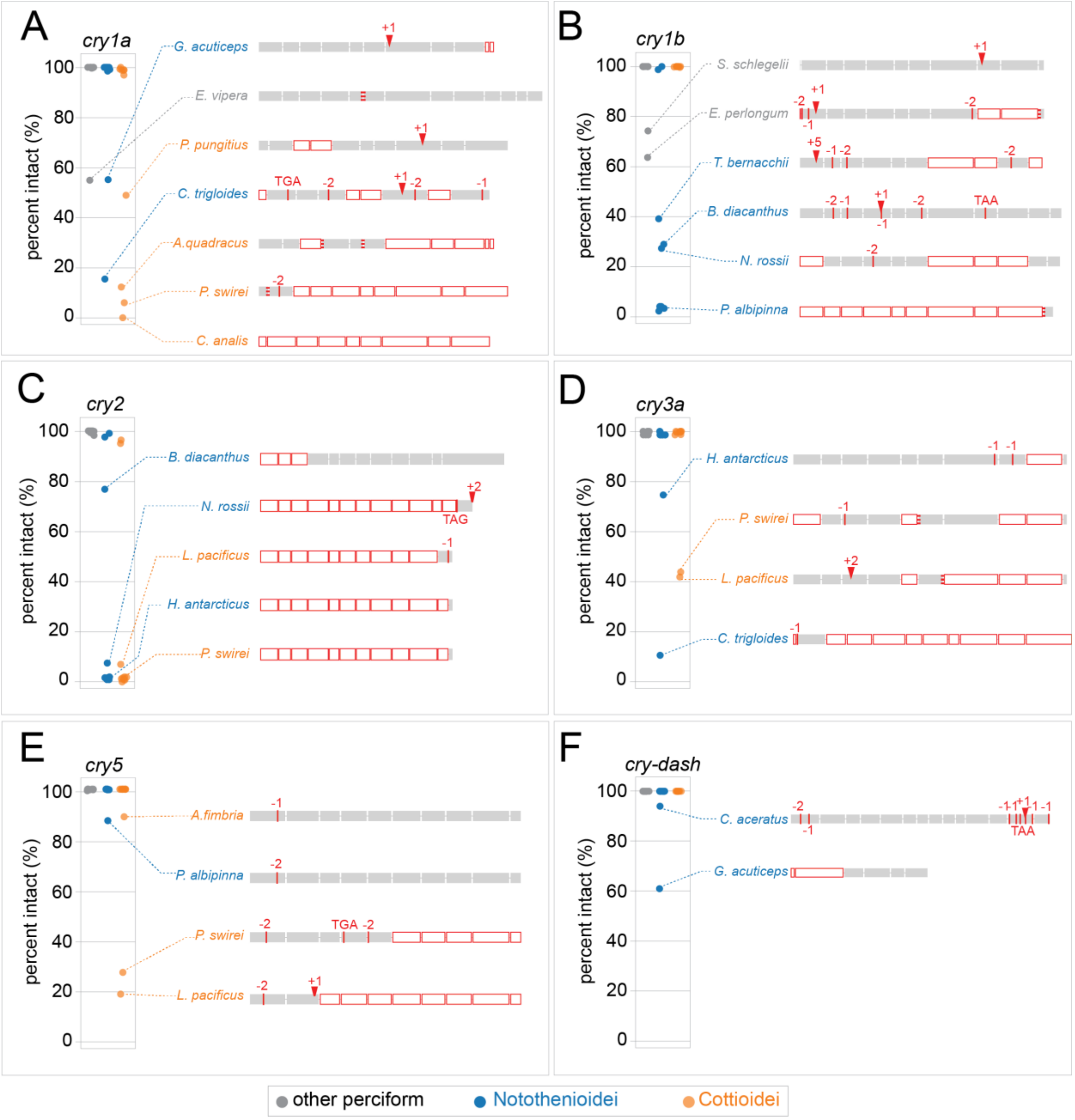


**Fig. S2 - Example mutational variants across *cryptochrome* genes**. A) *cry1a*, B) *cry1b*, C) *cry2*, D) *cry3a*, E) *cry5*, F) *cry-dash*. Plots show the maximum percentage of intact coding sequence across all transcript isoforms annotated via pairwise genome alignments to *S. lucioperca* and *S. aurata* reference genomes. Species are grouped as Notothenioidei (blue), Cottioidei (orange), or other Perciformes (gray). Representative mutations across each exon for select species are shown to the right. Exons are shaded gray; deleted or missing exons are white with a red outline. Truncating variants are labeled by their type (e.g., frameshifts as +1 or –1), and splice site mutations are marked with red dashed lines at exon boundaries.

bioRxiv preprint doi: https://doi.org/10.1101/2025.05.28.656707; this version posted May 31, 2025. The copyright holder for this preprint (which was not certified by peer review) is the author/funder, who has granted bioRxiv a license to display the preprint in perpetuity. It is made available under aCC-BY 4.0 International license.

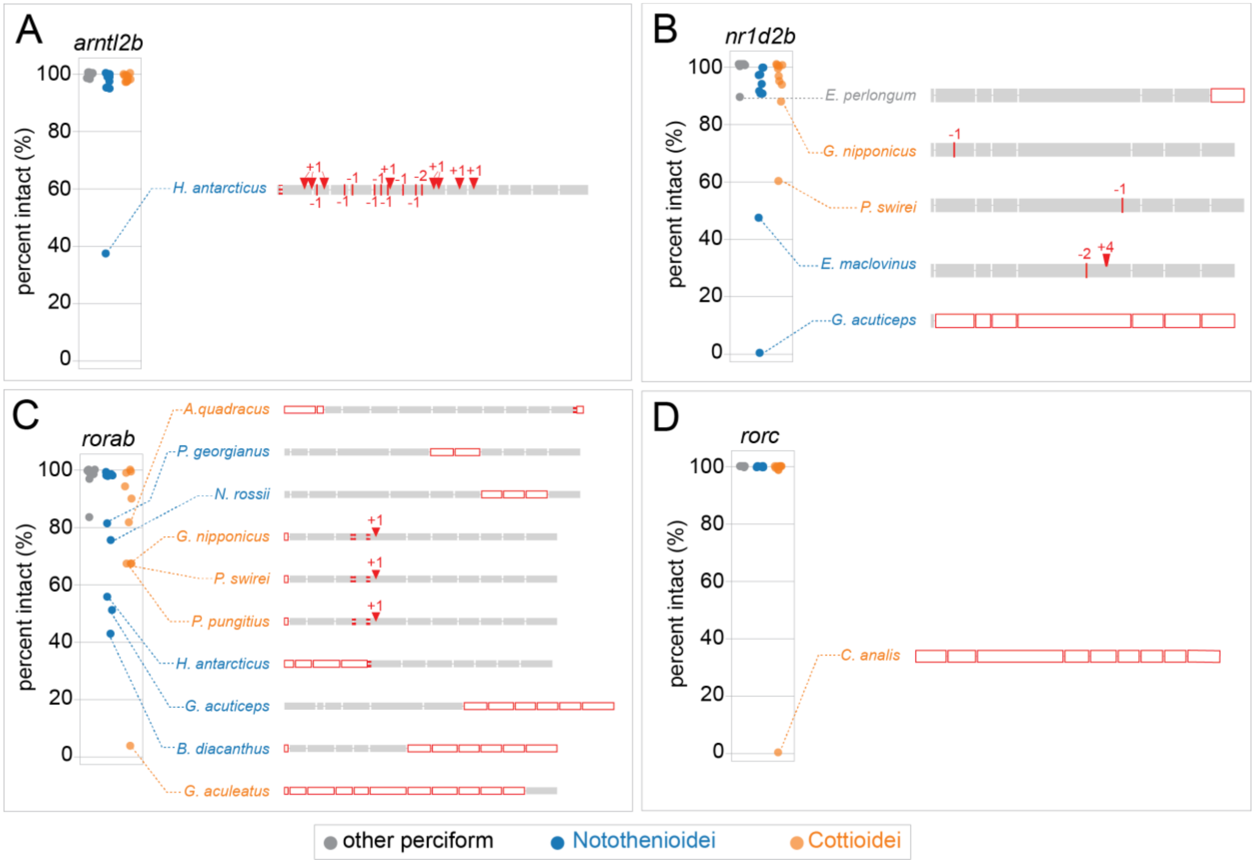


**Fig. S3 - Example mutational variants across assorted biological clock genes**. A) *arntl2b*, B) *nr1d2b*, C) *rorab*, D) *rorc*. Plots show the maximum percentage of intact coding sequence across all transcript isoforms annotated via pairwise genome alignments to *S. lucioperca* and *S. aurata* reference genomes. Species are grouped as Notothenioidei (blue), Cottioidei (orange), or other Perciformes (gray). Representative mutations across each exon for select species are shown to the right. Exons are shaded gray; deleted or missing exons are white with a red outline. Truncating variants are labeled by their type (e.g., frameshifts as +1 or –1), and splice site mutations are marked with red dashed lines at exon boundaries.

bioRxiv preprint doi: https://doi.org/10.1101/2025.05.28.656707; this version posted May 31, 2025. The copyright holder for this preprint (which was not certified by peer review) is the author/funder, who has granted bioRxiv a license to display the preprint in perpetuity. It is made available under aCC-BY 4.0 International license.

**Table S1** - Biological clock genes used in the analysis

| Gene name | Sander Ensembl ID | Sparus Ensembl ID |
|---|---|---|
| *arntl1a* | ENSSLUG00000025582 | ENSSAUG00010012423 |
| *arntl2a* | ENSSLUG00000025458 | ENSSAUG00010021136 |
| *arntl2b* | ENSSLUG00000023039 | ENSSAUG00010013586 |
| *clocka* | ENSSLUG00000011628 | ENSSAUG00010001441 |
| *clockb* | ENSSLUG00000011054 | ENSSAUG00010025019 |
| *cry-dash* | ENSSLUG00000012433 | ENSSAUG00010022353 |
| *cry1a* | ENSSLUG00000003675 | ENSSAUG00010012268 |
| *cry1b* | ENSSLUG00000002894 | ENSSAUG00010001264 |
| *cry2* | ENSSLUG00000025356 | ENSSAUG00010019549 |
| *cry3a* | ENSSLUG00000012954 | ENSSAUG00010004169 |
| *cry5* | ENSSLUG00000015034 | ENSSAUG00010015769 |
| *csnk1db* | ENSSLUG00000014906 | ENSSAUG00010004970 |
| *csnk1e* | ENSSLUG00000023267 | ENSSAUG00010009011 |
| *npas2* | ENSSLUG00000023767 | ENSSAUG00010018036 |
| *nr1d2a* | ENSSLUG00000011202 | ENSSAUG00010015973 |
| *nr1d2b* | ENSSLUG00000002539 | ENSSAUG00010013815 |
| *per1b* | ENSSLUG00000010714 | ENSSAUG00010014015 |
| *per2a* | ENSSLUG00000022765 | ENSSAUG00010010319 |
| *per2b* | ENSSLUG00000019172 | ENSSAUG00010027189 |
| *per3* | ENSSLUG00000011504 | ENSSAUG00010011519 |
| *roraa* | ENSSLUG00000015161 | ENSSAUG00010023974 |
| *rorab* | ENSSLUG00000016495 | ENSSAUG00010006455 |
| *rorb* | ENSSLUG00000022154 | ENSSAUG00010013250 |
| *rorc* | ENSSLUG00000010667 | ENSSAUG00010018260 |
| *rorca* | ENSSLUG00000007521 | ENSSAUG00010020966 |
| *rorcb* | ENSSLUG00000010091 | ENSSAUG00010018584 |
| *timeless* | ENSSLUG00000000306 | ENSSAUG00010024458 |

bioRxiv preprint doi: https://doi.org/10.1101/2025.05.28.656707; this version posted May 31, 2025. The copyright holder for this preprint (which was not certified by peer review) is the author/funder, who has granted bioRxiv a license to display the preprint in perpetuity. It is made available under aCC-BY 4.0 International license.

**Table S2** - Genome assemblies used in the analysis

| Order | Suborder | Family | Species | GenBank Accession | Length (Mb) | Scaffold count | Scaffold N50 (Mb) | Contig count | Contig N50 (Mb) |
|---|---|---|---|---|---|---|---|---|---|
| Perciformes | Cottioidei | Anoplopomatidae | Anoplopoma fimbria | GCA_027596085.2 | 653.5 | 7493 | 26.7 | 8212 | 2.6 |
| Perciformes | Cottioidei | Cottidae | Clinocottus analis | GCA_023055335.1 | 538.1 | 443 | 21.0 | 662 | 9.2 |
| Perciformes | Cottioidei | Cyclopteridae | Cyclopterus lumpus | GCA_009769545.1 | 572.9 | 49 | 23.9 | 396 | 5.0 |
| Perciformes | Cottioidei | Liparidae | Pseudoliparis swirei | GCA_029220125.1 | 626.4 | 199 | 25.7 | 1102 | 4.2 |
| Perciformes | Cottioidei | Gasterosteidae | Apeltes quadracus | GCA_048569185.1 | 475.9 | 215 | 18.5 | 339 | 10.5 |
| Perciformes | Cottioidei | Gasterosteidae | Pungitius pungitius | GCA_949316345.1 | 480.5 | 175 | 21.0 | 913 | 1.4 |
| Perciformes | Cottioidei | Gasterosteidae | Gasterosteus nipponicus | GCA_014132575.2 | 599.5 | 3095 | 17.6 | 3718 | 0.5 |
| Perciformes | Cottioidei | Gasterosteidae | Gasterosteus aculeatus | GCA_016920845.1 | 471.9 | 2937 | 20.5 | 6059 | 0.5 |
| Perciformes | Cottioidei | Stichaeidae | Cebidichthys violaceus | GCA_008087265.1 | 593.0 | 467 | 6.7 | 505 | 5.5 |
| Perciformes | Cottioidei | Zoarcidae | Lycodopsis pacificus | GCA_028022725.1 | 646.4 | 109 | 27.7 | 475 | 4.7 |
| Perciformes | Notothenioidei | Artedidraconidae | Pogonophryne albipinna | GCA_028583405.1 | 1074.5 | 1111 | 41.8 | 3897 | 1.0 |
| Perciformes | Notothenioidei | Bathydraconidae | Gymnodraco acuticeps | GCA_902827175.1 | 996.9 | 2618 | 1.9 | 4183 | 0.5 |
| Perciformes | Notothenioidei | Bovichtidae | Cottoperca trigloides | GCA_900634415.1 | 609.4 | 322 | 25.2 | 766 | 6.3 |
| Perciformes | Notothenioidei | Bovichtidae | Bovichtus diacanthus | GCA_943590825.1 | 641.8 | 12443 | 7.3 | 44874 | 0.0 |
| Perciformes | Notothenioidei | Channichthyidae | Pseudochaenichthys georgianus | GCA_902827115.2 | 1026.2 | 1562 | 42.8 | 4129 | 0.7 |
| Perciformes | Notothenioidei | Channichthyidae | Chaenocephalus aceratus | GCA_023974075.1 | 1065.6 | 3134 | 33.5 | 3856 | 1.5 |
| Perciformes | Notothenioidei | Eleginopidae | Eleginops maclovinus | GCA_036324505.1 | 606.3 | 26 | 26.7 | 406 | 7.6 |
| Perciformes | Notothenioidei | Harpagiferidae | Harpagifer antarcticus | GCA_902827135.1 | 941.5 | 1541 | 5.0 | 2579 | 1.1 |
| Perciformes | Notothenioidei | Nototheniidae | Trematomus bernacchii | GCA_902827165.1 | 867.1 | 864 | 8.8 | 1793 | 1.4 |
| Perciformes | Notothenioidei | Nototheniidae | Notothenia rossii | GCA_949606895.1 | 1042.9 | 943 | 89.7 | 5100 | 0.4 |
| Perciformes | Percoidei | Percidae | Gymnocephalus cernua | GCA_023631565.1 | 904.3 | 156 | 38.2 | 583 | 10.9 |
| Perciformes | Percoidei | Percidae | Sander lucioperca | GCA_008315115.1 | 900.5 | 1312 | 4.9 | 1347 | 4.7 |
| Spariformes | N/A | Sparidae | Sparus aurata | GCA_900880675.1 | 833.2 | 175 | 35.8 | 1223 | 2.9 |
| Perciformes | Scorpaenoidei | Scorpaenidae | Pterois miles | GCA_947000775.1 | 902.4 | N/A | N/A | 660 | 14.5 |
| Perciformes | Percoidei | Percidae | Perca flavescens | GCA_004354835.1 | 877.4 | 267 | 37.4 | 1096 | 4.3 |
| Perciformes | Percoidei | Percidae | Etheostoma perlongum | GCA_026937815.1 | 788.7 | 1179 | 30.8 | 2095 | 2.3 |
| Perciformes | Scorpaenoidei | Sebastidae | Sebastes schlegelii | GCA_014673565.1 | 848.0 | 1663 | 7.9 | 2110 | 5.5 |
| Perciformes | Scorpaenoidei | Synanceiidae | Synanceia verrucosa | GCA_029721515.1 | 691.9 | 1532 | 27.1 | 2325 | 12.0 |
| Perciformes | Serranoidei | Serranidae | Epinephelus lanceolatus | GCA_041903045.1 | 1089.4 | 176 | 45.8 | 183 | 44.4 |
| Perciformes | Serranoidei | Serranidae | Hypoplectrus puella | GCA_964304535.1 | 686.8 | 253 | 26.1 | 268 | 23.2 |
| Perciformes | Triglioidei | Triglidae | Chelidonichthys spinosus | GCA_029853015.1 | 624.7 | 293 | 28.1 | 406 | 13.8 |
| Perciformes | Percoidei | Trachinidae | Echiichthys vipera | GCA_963691815.1 | 800.4 | 133 | 33.0 | 739 | 2.6 |
| Perciformes | Serranoidei | Serranidae | Epinephelus cyanopodus | GCA_026686955.1 | 998.8 | 106 | 42.0 | 458 | 5.9 |
| Perciformes | Serranoidei | Serranidae | Centropristis striata | GCA_030273125.1 | 926.0 | 92 | 39.0 | 433 | 9.5 |
| Scombriformes | N/A | Scombridae | Thunnus maccoyii | GCA_910596095.1 | 782.4 | 57 | 33.8 | 152 | 26.8 |
| Pleuronectiformes | N/A | Pleuronectidae | Hippoglossus hippoglossus | GCA_009819705.1 | 596.8 | 56 | 26.3 | 346 | 7.0 |
| Cichliformes | N/A | Cichlidae | Oreochromis aureus | GCA_013358895.1 | 1000.0 | 303 | 40.7 | 1025 | 4.2 |
| Atheriniformes | N/A | Melanotaeniidae | Melanotaenia boesemani | GCA_017639745.1 | 865.6 | 92 | 37.9 | 532 | 9.3 |
| Acanthuriformes | N/A | Moronidae | Dicentrarchus labrax | GCA_905237075.1 | 695.9 | 302 | 29.9 | 574 | 12.7 |

bioRxiv preprint doi: https://doi.org/10.1101/2025.05.28.656707; this version posted May 31, 2025. The copyright holder for this preprint (which was not certified by peer review) is the author/funder, who has granted bioRxiv a license to display the preprint in perpetuity. It is made available under aCC-BY 4.0 International license.

**Table S3** - Relaxed selection across biological clock genes

| | | Reference genome | | | |
|---|---|---|---|---|---|
| | | S. lucioperca | | S. auratus | |
| Foreground branches | gene | K | p.adj | K | p.adj |
| Notothenioidei + Cottioidei | *arntl2a* | 0.82 | 3.76E-02 | 0.56 | 1.59E-02 |
| Notothenioidei + Cottioidei | *clocka* | 0.16 | 2.75E-03 | 0.72 | 5.01E-07 |
| Notothenioidei + Cottioidei | *cry1a* | 0.47 | 3.11E-06 | 0.45 | 2.25E-07 |
| Notothenioidei + Cottioidei | *cry5* | 0.85 | 2.17E-02 | 0.87 | 3.64E-02 |
| Notothenioidei + Cottioidei | *per1b* | 0.82 | 5.29E-08 | 0.00 | 4.74E-08 |
| Notothenioidei + Cottioidei | *per2a* | 0.51 | 6.15E-08 | 0.36 | 7.40E-04 |
| Notothenioidei + Cottioidei | *rorb* | 0.53 | 4.30E-05 | 0.50 | 1.80E-02 |
| Notothenioidei + Cottioidei | *rorc* | 0.79 | 1.71E-03 | 0.70 | 8.73E-05 |
| Notothenioidei + Cottioidei | *rorca* | 0.00 | 9.03E-03 | 0.25 | 2.47E-03 |
| Notothenioidei | *arntl2b* | 0.63 | 7.80E-02 | 0.50 | 2.47E-02 |
| Notothenioidei | *npas2* | 15.78 | 8.28E-02 | 8.40 | 2.51E-02 |
| Notothenioidei | *rorc* | 0.78 | 3.74E-02 | 0.72 | 2.80E-03 |
| Cottioidei | *cry1b* | 1.19 | 4.11E-02 | 1.16 | 6.77E-02 |
| Cottioidei | *clocka* | 0.70 | 1.52E-03 | 0.79 | 4.12E-04 |
| Cottioidei | *cry3a* | 0.71 | 1.51E-08 | 0.68 | 3.77E-05 |
| Cottioidei | *rorab* | 0.67 | 1.64E-03 | 0.83 | 5.27E-02 |
| Cottioidei | *cry1a* | 0.43 | 2.33E-05 | 0.69 | 1.07E-04 |
| Cottioidei | *rorca* | 0.63 | 6.35E-04 | 0.47 | 4.12E-04 |
| Cottioidei | *arntl2a* | 0.36 | 2.33E-05 | 0.14 | 1.09E-05 |

bioRxiv preprint doi: https://doi.org/10.1101/2025.05.28.656707; this version posted May 31, 2025. The copyright holder for this preprint (which was not certified by peer review) is the author/funder, who has granted bioRxiv a license to display the preprint in perpetuity. It is made available under aCC-BY 4.0 International license.

**Table S4** – Species location data. Latitude and depth data from Aquamaps (88).

| Species | Median Center Lat | Mean Center Lat | Median Depth | Mean Depth | Max Depth | Observation Count | losses |
|---|---|---|---|---|---|---|---|
| Anoplopoma_fimbria | 54.25 | 52.01 | 454 | 1093.98 | 7119 | 793 | 0 |
| Apeltes_quadracus | 45.25 | 44.57 | 24 | 58.82 | 1843 | 135 | 5 |
| Bovichtus_diacanthus | -38.75 | -38.83 | 2952 | 3115.00 | 5059 | 6 | 1 |
| Cebidichthys_violaceus | 37.25 | 37.55 | 222 | 346.91 | 1365 | 33 | 1 |
| Centropristis_striata | 36.25 | 35.55 | 26 | 209.85 | 4495 | 212 | 0 |
| Chaenocephalus_aceratus | -60.75 | -59.11 | 460 | 879.96 | 3724 | 69 | 6 |
| Chelidonichthys_spinosus | 33.75 | 30.58 | 77 | 453.64 | 7119 | 56 | 0 |
| Clinocottus_analis | 33.75 | 33.35 | 417 | 708.67 | 4779 | 63 | 3 |
| Cottoperca_trigloides | -46.75 | -46.98 | 102 | 162.29 | 1552 | 179 | 4 |
| Cyclopterus_lumpus | 54.25 | 54.36 | 101 | 325.54 | 4784 | 1624 | 2 |
| Echiichthys_vipera | 52.25 | 50.01 | 41 | 176.18 | 4888 | 343 | 0 |
| Eleginops_maclovinus | -45.75 | -45.57 | 49 | 133.96 | 2341 | 68 | 1 |
| Epinephelus_lanceolatus | -10.75 | -6.31 | 262 | 1226.10 | 7470 | 229 | 0 |
| Epinephelus_cyanopodus | -14.75 | -8.11 | 474 | 926.55 | 4327 | 121 | 0 |
| Gasterosteus_aculeatus | 53.25 | 52.06 | 44 | 190.45 | 5930 | 1569 | 5 |
| Gymnodraco_acuticeps | -65.25 | -65.80 | 842 | 1539.42 | 4237 | 207 | 5 |
| Hypoplectrus_puella | 19.25 | 19.80 | 337 | 564.84 | 2588 | 129 | 0 |
| Lycodes_pacificus | 49.75 | 48.15 | 142.5 | 411.06 | 5033 | 172 | 4 |
| Notothenia_rossii | -54.25 | -55.02 | 459.5 | 1008.90 | 4612 | 82 | 3 |
| Pseudochaenichthys_georgianus | -60.25 | -58.44 | 465 | 1023.67 | 3944 | 51 | 4 |
| Pseudoliparis_swirei | 39.5 | 41.13 | 6162 | 6242.00 | 6864 | 4 | 7 |
| Pterois_miles | 2.25 | -1.08 | 190.5 | 529.71 | 4082 | 156 | 0 |
| Pungitius_pungitius | 55.25 | 55.64 | 20 | 46.29 | 2189 | 678 | 4 |
| Sebastes_schlegelii | 37.25 | 38.16 | 33 | 89.53 | 720 | 45 | 0 |
| Synanceia_verrucosa | -6.75 | -2.41 | 422 | 953.62 | 6286 | 249 | 0 |
| Trematomus_bernacchii | -66.25 | -67.50 | 350 | 705.87 | 4237 | 124 | 4 |

bioRxiv preprint doi: https://doi.org/10.1101/2025.05.28.656707; this version posted May 31, 2025. The copyright holder for this preprint (which was not certified by peer review) is the author/funder, who has granted bioRxiv a license to display the preprint in perpetuity. It is made available under aCC-BY 4.0 International license.

**Table S5** - Breakdown of global gene status (TOGA) by reference genome

| Reference | Species | I+PI | L+UL | I | PI | L | UL | M | PM | PG |
|---|---|---|---|---|---|---|---|---|---|---|
| sparus_aurata | anoplopoma_fimbria | 72.3 | 26.5 | 71.2 | 1.0 | 11.6 | 14.9 | 0.7 | 0.3 | 0.3 |
| sander_lucioperca | anoplopoma_fimbria | 69.1 | 27.3 | 68.3 | 0.8 | 9.7 | 17.6 | 2.0 | 0.4 | 1.2 |
| merged | anoplopoma_fimbria | 76.1 | 20.7 | 75.3 | 0.8 | 9.5 | 11.2 | 1.9 | 0.3 | 1.0 |
| sparus_aurata | apeltes_quadracus | 62.8 | 34.6 | 61.9 | 0.9 | 18.0 | 16.6 | 1.5 | 0.3 | 0.8 |
| sander_lucioperca | apeltes_quadracus | 59.6 | 32.3 | 59.0 | 0.5 | 13.9 | 18.4 | 3.9 | 0.4 | 3.8 |
| merged | apeltes_quadracus | 66.7 | 26.8 | 66.1 | 0.6 | 14.1 | 12.7 | 3.7 | 0.4 | 2.4 |
| sparus_aurata | bovichtus_diacanthus | 67.6 | 21.9 | 60.8 | 6.8 | 12.8 | 9.1 | 5.9 | 1.8 | 2.8 |
| sander_lucioperca | bovichtus_diacanthus | 66.7 | 20.0 | 60.2 | 6.4 | 8.2 | 11.9 | 7.9 | 1.8 | 3.3 |
| merged | bovichtus_diacanthus | 73.0 | 15.4 | 66.1 | 6.9 | 8.7 | 6.7 | 7.4 | 1.5 | 2.7 |
| sparus_aurata | cebidichthys_violaceus | 72.7 | 23.3 | 71.0 | 1.7 | 13.1 | 10.3 | 2.4 | 0.4 | 1.1 |
| sander_lucioperca | cebidichthys_violaceus | 68.5 | 24.1 | 67.2 | 1.3 | 10.3 | 13.8 | 4.3 | 0.5 | 2.6 |
| merged | cebidichthys_violaceus | 75.4 | 18.7 | 74.0 | 1.3 | 10.9 | 7.8 | 3.6 | 0.5 | 1.9 |
| sparus_aurata | centropristis_striata | 79.8 | 19.3 | 78.8 | 1.0 | 9.7 | 9.6 | 0.5 | 0.2 | 0.2 |
| sander_lucioperca | centropristis_striata | 74.7 | 22.1 | 73.8 | 0.9 | 8.4 | 13.7 | 1.6 | 0.4 | 1.3 |
| merged | centropristis_striata | 82.2 | 15.1 | 81.3 | 0.9 | 7.9 | 7.2 | 1.4 | 0.3 | 1.0 |
| sparus_aurata | chaenocephalus_aceratus | 53.0 | 40.0 | 51.6 | 1.5 | 18.9 | 21.2 | 3.5 | 0.9 | 2.5 |
| sander_lucioperca | chaenocephalus_aceratus | 51.8 | 39.3 | 50.4 | 1.3 | 16.4 | 23.0 | 4.8 | 1.0 | 3.2 |
| merged | chaenocephalus_aceratus | 57.2 | 35.4 | 55.7 | 1.5 | 17.8 | 17.6 | 4.5 | 0.8 | 2.2 |
| sparus_aurata | chelidonichthys_spinosus | 70.3 | 28.7 | 69.5 | 0.9 | 13.6 | 15.1 | 0.4 | 0.4 | 0.1 |
| sander_lucioperca | chelidonichthys_spinosus | 66.5 | 28.6 | 65.8 | 0.7 | 11.5 | 17.1 | 2.6 | 0.5 | 1.9 |
| merged | chelidonichthys_spinosus | 73.6 | 22.5 | 72.9 | 0.7 | 11.4 | 11.1 | 2.3 | 0.4 | 1.3 |
| sparus_aurata | clinocottus_analis | 74.2 | 23.9 | 73.3 | 0.9 | 14.2 | 9.7 | 1.0 | 0.3 | 0.7 |
| sander_lucioperca | clinocottus_analis | 70.2 | 23.8 | 69.6 | 0.7 | 11.0 | 12.9 | 2.9 | 0.4 | 2.6 |
| merged | clinocottus_analis | 77.7 | 17.6 | 77.0 | 0.7 | 10.8 | 6.8 | 2.6 | 0.4 | 1.8 |
| sparus_aurata | cottoperca_gobio | 64.1 | 31.4 | 63.0 | 1.1 | 15.3 | 16.2 | 2.9 | 0.5 | 1.1 |
| sander_lucioperca | cottoperca_gobio | 62.6 | 31.0 | 61.6 | 1.0 | 12.3 | 18.7 | 4.0 | 0.7 | 1.7 |
| merged | cottoperca_gobio | 68.8 | 25.7 | 67.7 | 1.1 | 12.8 | 12.9 | 3.6 | 0.6 | 1.3 |
| sparus_aurata | cyclopterus_lumpus | 70.0 | 27.8 | 69.1 | 0.8 | 16.1 | 11.8 | 1.4 | 0.3 | 0.5 |
| sander_lucioperca | cyclopterus_lumpus | 66.7 | 27.0 | 66.0 | 0.8 | 12.5 | 14.5 | 3.7 | 0.5 | 2.1 |
| merged | cyclopterus_lumpus | 73.9 | 20.9 | 73.1 | 0.8 | 12.6 | 8.4 | 3.3 | 0.4 | 1.5 |
| sparus_aurata | dicentrarchus_labrax | 81.5 | 18.0 | 80.4 | 1.1 | 7.9 | 10.1 | 0.3 | 0.2 | 0.1 |
| sander_lucioperca | dicentrarchus_labrax | 74.4 | 22.7 | 73.5 | 0.8 | 8.5 | 14.2 | 1.4 | 0.4 | 1.2 |
| merged | dicentrarchus_labrax | 82.0 | 15.4 | 81.2 | 0.8 | 7.8 | 7.5 | 1.3 | 0.3 | 1.0 |
| sparus_aurata | echiichthys_vipera | 76.2 | 22.8 | 75.0 | 1.2 | 12.8 | 10.1 | 0.4 | 0.4 | 0.2 |
| sander_lucioperca | echiichthys_vipera | 72.8 | 23.3 | 71.8 | 1.0 | 9.7 | 13.6 | 1.9 | 0.4 | 1.7 |
| merged | echiichthys_vipera | 80.0 | 16.5 | 79.0 | 1.0 | 9.3 | 7.2 | 1.8 | 0.4 | 1.3 |
| sparus_aurata | eleginops_maclovinus | 68.0 | 29.7 | 67.2 | 0.8 | 15.0 | 14.7 | 1.3 | 0.4 | 0.6 |

bioRxiv preprint doi: https://doi.org/10.1101/2025.05.28.656707; this version posted May 31, 2025. The copyright holder for this preprint (which was not certified by peer review) is the author/funder, who has granted bioRxiv a license to display the preprint in perpetuity. It is made available under aCC-BY 4.0 International license.

| | | | | | | | | | | |
|---|---|---|---|---|---|---|---|---|---|---|
| sander_lucioperca | eleginops_maclovinus | 66.0 | 28.6 | 65.2 | 0.8 | 11.5 | 17.2 | 2.7 | 0.5 | 2.1 |
| merged | eleginops_maclovinus | 66.7 | 30.9 | 65.8 | 0.9 | 9.4 | 21.6 | 1.3 | 0.2 | 0.8 |
| sander_lucioperca | epinephalus_cyanopodus | 66.7 | 31.0 | 65.8 | 0.9 | 9.4 | 21.6 | 1.3 | 0.2 | 0.8 |
| sparus_aurata | epinephalus_cyanopodus | 71.1 | 28.3 | 70.2 | 0.9 | 9.7 | 18.6 | 0.3 | 0.2 | 0.1 |
| merged | epinephalus_cyanopodus | 73.3 | 24.6 | 72.4 | 0.9 | 9.4 | 15.3 | 1.2 | 0.2 | 0.7 |
| sparus_aurata | epinephelus_lanceolatus | 78.4 | 19.7 | 75.9 | 2.5 | 8.9 | 10.7 | 1.4 | 0.3 | 0.1 |
| sander_lucioperca | epinephelus_lanceolatus | 74.1 | 21.3 | 72.1 | 2.0 | 7.5 | 13.7 | 3.2 | 0.5 | 1.0 |
| merged | epinephelus_lanceolatus | 81.1 | 14.7 | 79.0 | 2.0 | 7.2 | 7.5 | 3.0 | 0.4 | 0.8 |
| sparus_aurata | etheostoma_perlongum | 58.4 | 38.9 | 57.3 | 1.1 | 14.4 | 24.5 | 1.9 | 0.4 | 0.5 |
| sander_lucioperca | etheostoma_perlongum | 62.5 | 35.5 | 61.3 | 1.3 | 9.1 | 26.5 | 1.2 | 0.4 | 0.3 |
| merged | etheostoma_perlongum | 67.5 | 30.8 | 66.2 | 1.3 | 9.6 | 21.2 | 1.2 | 0.3 | 0.3 |
| sparus_aurata | gasterosteus_aculeatus | 65.5 | 29.1 | 64.3 | 1.3 | 16.0 | 13.1 | 4.1 | 0.4 | 0.9 |
| sander_lucioperca | gasterosteus_aculeatus | 62.9 | 27.4 | 61.9 | 1.0 | 12.2 | 15.2 | 5.5 | 0.6 | 3.5 |
| merged | gasterosteus_aculeatus | 70.1 | 21.9 | 69.0 | 1.1 | 12.3 | 9.6 | 5.3 | 0.5 | 2.2 |
| sparus_aurata | gasterosteus_nipponicus | 56.9 | 39.6 | 55.5 | 1.4 | 17.7 | 21.8 | 2.0 | 0.4 | 1.2 |
| sander_lucioperca | gasterosteus_nipponicus | 54.6 | 36.9 | 53.5 | 1.1 | 14.1 | 22.8 | 4.1 | 0.6 | 3.8 |
| merged | gasterosteus_nipponicus | 61.3 | 31.8 | 60.2 | 1.2 | 14.4 | 17.4 | 4.0 | 0.5 | 2.4 |
| sparus_aurata | gymnocephalus_cernua | 78.3 | 20.6 | 77.1 | 1.2 | 10.6 | 10.1 | 0.6 | 0.3 | 0.2 |
| sander_lucioperca | gymnocephalus_cernua | 83.2 | 16.0 | 82.1 | 1.1 | 4.6 | 11.4 | 0.5 | 0.2 | 0.1 |
| merged | gymnocephalus_cernua | 88.0 | 11.4 | 86.8 | 1.2 | 4.5 | 6.8 | 0.4 | 0.2 | 0.1 |
| sparus_aurata | gymnodraco_acuticeps | 63.4 | 29.1 | 61.2 | 2.2 | 14.1 | 15.0 | 3.3 | 1.0 | 3.2 |
| sander_lucioperca | gymnodraco_acuticeps | 61.5 | 28.7 | 59.6 | 1.9 | 11.4 | 17.3 | 5.0 | 1.3 | 3.6 |
| merged | gymnodraco_acuticeps | 67.9 | 23.7 | 65.7 | 2.2 | 12.0 | 11.6 | 4.7 | 1.1 | 2.7 |
| sparus_aurata | hippoglossus_hippoglossus | 71.5 | 25.3 | 70.8 | 0.7 | 15.2 | 10.2 | 2.1 | 0.3 | 0.7 |
| sander_lucioperca | hippoglossus_hippoglossus | 67.3 | 25.5 | 66.8 | 0.5 | 12.2 | 13.3 | 4.4 | 0.5 | 2.4 |
| merged | hippoglossus_hippoglossus | 74.9 | 18.9 | 74.4 | 0.5 | 11.9 | 7.0 | 4.1 | 0.4 | 1.7 |
| sparus_aurata | hypoplectrus_puella | 74.7 | 20.5 | 66.9 | 7.8 | 10.5 | 9.9 | 3.5 | 0.4 | 0.9 |
| sander_lucioperca | hypoplectrus_puella | 70.4 | 21.3 | 64.1 | 6.3 | 8.6 | 12.7 | 4.7 | 0.6 | 3.1 |
| merged | hypoplectrus_puella | 77.7 | 15.1 | 70.7 | 7.0 | 8.3 | 6.8 | 4.5 | 0.6 | 2.1 |
| sparus_aurata | lycodes_pacificus | 76.9 | 21.7 | 75.8 | 1.1 | 12.0 | 9.7 | 0.9 | 0.2 | 0.3 |
| sander_lucioperca | lycodes_pacificus | 72.1 | 23.8 | 71.2 | 0.9 | 10.2 | 13.7 | 2.2 | 0.4 | 1.5 |
| merged | lycodes_pacificus | 79.4 | 17.0 | 78.5 | 0.9 | 9.8 | 7.2 | 2.1 | 0.3 | 1.2 |
| sparus_aurata | melanotaenia_boesmani | 73.1 | 24.2 | 72.2 | 0.8 | 14.3 | 9.9 | 1.6 | 0.3 | 0.9 |
| sander_lucioperca | melanotaenia_boesmani | 66.8 | 25.1 | 66.3 | 0.6 | 12.4 | 12.7 | 3.5 | 0.6 | 4.0 |
| merged | melanotaenia_boesmani | 75.4 | 18.6 | 74.7 | 0.7 | 12.0 | 6.6 | 3.3 | 0.5 | 2.3 |
| sparus_aurata | notothenia_rossii | 73.5 | 23.6 | 69.7 | 3.8 | 12.3 | 11.2 | 2.2 | 0.3 | 0.5 |
| sander_lucioperca | notothenia_rossii | 69.3 | 25.0 | 65.9 | 3.4 | 10.3 | 14.7 | 3.6 | 0.4 | 1.6 |
| merged | notothenia_rossii | 76.6 | 18.9 | 72.8 | 3.7 | 10.5 | 8.5 | 3.0 | 0.4 | 1.1 |
| sparus_aurata | oreochromis_aureus | 75.6 | 21.7 | 74.7 | 0.9 | 12.0 | 9.7 | 1.5 | 0.3 | 0.9 |
| sander_lucioperca | oreochromis_aureus | 68.8 | 23.5 | 68.1 | 0.7 | 10.5 | 12.9 | 3.7 | 0.5 | 3.6 |

bioRxiv preprint doi: https://doi.org/10.1101/2025.05.28.656707; this version posted May 31, 2025. The copyright holder for this preprint (which was not certified by peer review) is the author/funder, who has granted bioRxiv a license to display the preprint in perpetuity. It is made available under aCC-BY 4.0 International license.

| | | | | | | | | | | |
|---|---|---|---|---|---|---|---|---|---|---|
| merged | oreochromis_aureus | 77.4 | 16.5 | 76.7 | 0.7 | 10.1 | 6.4 | 3.5 | 0.4 | 2.2 |
| sparus_aurata | perca_flavescens | 75.9 | 22.5 | 74.8 | 1.0 | 10.9 | 11.7 | 1.0 | 0.3 | 0.3 |
| sander_lucioperca | perca_flavescens | 82.5 | 17.0 | 81.5 | 1.1 | 4.4 | 12.6 | 0.2 | 0.2 | 0.1 |
| merged | perca_flavescens | 87.1 | 12.4 | 86.0 | 1.1 | 4.4 | 8.0 | 0.2 | 0.2 | 0.1 |
| sparus_aurata | pogonophryne_albipinna | 73.0 | 24.2 | 71.3 | 1.7 | 12.9 | 11.2 | 1.6 | 0.4 | 0.8 |
| sander_lucioperca | pogonophryne_albipinna | 69.3 | 25.3 | 67.8 | 1.5 | 10.9 | 14.4 | 2.9 | 0.5 | 1.9 |
| merged | pogonophryne_albipinna | 76.4 | 19.2 | 74.8 | 1.6 | 11.2 | 8.0 | 2.7 | 0.4 | 1.3 |
| sparus_aurata | pseudochaenichthys_georgianus | 66.0 | 26.5 | 64.1 | 1.9 | 14.3 | 12.2 | 4.3 | 0.9 | 2.2 |
| sander_lucioperca | pseudochaenichthys_georgianus | 64.0 | 26.5 | 62.3 | 1.7 | 11.6 | 14.9 | 5.3 | 1.0 | 3.2 |
| merged | pseudochaenichthys_georgianus | 70.4 | 21.5 | 68.5 | 1.9 | 12.4 | 9.1 | 5.1 | 0.7 | 2.2 |
| sparus_aurata | pseudoliparis_swirei | 63.8 | 31.6 | 62.8 | 0.9 | 20.4 | 11.2 | 3.1 | 0.4 | 1.0 |
| sander_lucioperca | pseudoliparis_swirei | 61.4 | 28.3 | 60.6 | 0.8 | 14.5 | 13.9 | 6.2 | 0.7 | 3.5 |
| merged | pseudoliparis_swirei | 68.5 | 23.5 | 67.7 | 0.8 | 15.5 | 8.0 | 5.4 | 0.5 | 2.0 |
| sparus_aurata | pterois_miles | 73.9 | 24.8 | 73.0 | 0.9 | 12.3 | 12.5 | 0.7 | 0.3 | 0.3 |
| sander_lucioperca | pterois_miles | 70.6 | 26.0 | 69.9 | 0.7 | 10.2 | 15.8 | 1.8 | 0.3 | 1.2 |
| merged | pterois_miles | 77.7 | 19.2 | 77.0 | 0.7 | 9.8 | 9.5 | 1.7 | 0.3 | 1.0 |
| sparus_aurata | pungitius_pungitius | 71.6 | 25.8 | 70.3 | 1.3 | 15.7 | 10.1 | 1.7 | 0.3 | 0.7 |
| sander_lucioperca | pungitius_pungitius | 67.1 | 25.4 | 66.1 | 1.0 | 12.3 | 13.1 | 3.7 | 0.5 | 3.3 |
| merged | pungitius_pungitius | 75.2 | 19.0 | 74.2 | 1.1 | 12.2 | 6.8 | 3.4 | 0.4 | 2.1 |
| sparus_aurata | sander_lucioperca | 73.1 | 25.4 | 72.0 | 1.1 | 10.7 | 14.7 | 0.7 | 0.4 | 0.5 |
| sparus_aurata | sebastes_schlegelii | 74.9 | 23.2 | 73.8 | 1.1 | 10.4 | 12.8 | 1.3 | 0.3 | 0.3 |
| sander_lucioperca | sebastes_schlegelii | 70.8 | 25.4 | 69.9 | 0.9 | 9.3 | 16.1 | 2.4 | 0.4 | 1.1 |
| merged | sebastes_schlegelii | 77.8 | 18.8 | 76.8 | 1.0 | 9.1 | 9.7 | 2.2 | 0.3 | 0.9 |
| sparus_aurata | synanceia_verrucosa | 74.6 | 23.8 | 73.8 | 0.9 | 14.4 | 9.3 | 0.8 | 0.5 | 0.3 |
| sander_lucioperca | synanceia_verrucosa | 70.5 | 23.4 | 70.0 | 0.5 | 10.9 | 12.5 | 3.3 | 0.5 | 2.3 |
| merged | synanceia_verrucosa | 77.8 | 17.1 | 77.3 | 0.5 | 10.9 | 6.2 | 3.0 | 0.5 | 1.6 |
| sparus_aurata | thunnus_maccoyii | 79.8 | 19.0 | 79.0 | 0.9 | 9.4 | 9.6 | 0.7 | 0.3 | 0.2 |
| sander_lucioperca | thunnus_maccoyii | 74.1 | 22.3 | 73.4 | 0.8 | 9.0 | 13.3 | 2.0 | 0.3 | 1.3 |
| merged | thunnus_maccoyii | 81.7 | 15.0 | 81.0 | 0.7 | 8.3 | 6.7 | 1.9 | 0.3 | 1.1 |
| sparus_aurata | trematomus_bernacchii | 69.0 | 26.9 | 67.9 | 1.1 | 12.9 | 13.9 | 2.1 | 0.6 | 1.5 |
| sander_lucioperca | trematomus_bernacchii | 66.2 | 27.7 | 65.1 | 1.1 | 11.0 | 16.7 | 3.4 | 0.6 | 2.1 |
| merged | trematomus_bernacchii | 73.0 | 21.6 | 71.9 | 1.1 | 11.1 | 10.5 | 3.3 | 0.5 | 1.6 |
